# A Simple Combination of Active and Intelligent Packaging Based on Garlic Extract and Indicator Solution in Extending and Monitoring the Meat Quality Stored at Cold Temperature

**DOI:** 10.3390/foods11101495

**Published:** 2022-05-20

**Authors:** Andi Dirpan, Muspirah Djalal, Andi Fadiah Ainani

**Affiliations:** Department of Agricultural Technology, Hasanuddin University, Makassar 90245, Indonesia; muspirah_djalal@agri.unhas.ac.id (M.D.); andifadiah97@gmail.com (A.F.A.)

**Keywords:** smart packaging, meat, garlic oil, shelf life

## Abstract

Safety and quality, as the major concerns of meat, are highly dependent on the ingredients and packaging techniques used. A basic combination of active and intelligent packaging is believed to be capable of preserving product quality, extending shelf life, and monitoring product deterioration. Therefore, this study aimed to extend and monitor the beef quality at cold temperatures (4 ± 1 °C). The active packaging applied garlic extract (0%, 15%, and 20% (*w*/*w*)) to release anti-microbial agents. Meanwhile, the intelligent paper applied a combination of bromothymol blue (BTB) and phenol red (PR) solutions at pH 5.00. The results showed that beef packed without the addition of garlic extract had already deteriorated on the 6th day of storage while, with the addition of garlic extract (15% and 20%) rotted on the 12th day. The intelligent indication label’s color profile changed from dark yellow (fresh), to reddish-yellow (to be consumed immediately), to faded red (rotten). The color change of the intelligent indicator label in response to all meat deterioration criteria demonstrates a linear correlation for determining the extent of rottenness during storage. Therefore, this simple combination of active paper and intelligent indicator can be used to extend the shelf life and monitor meat quality.

## 1. Introduction

Meat is one of the most essential nutrients, containing high levels of easily digestible protein, calorie-dense fat, vitamins, and other micronutrients. Its high nutritional value corresponds to its rising demand as the world’s most significant food product during the past several years [1]. The high demand should be fulfilled with the availability and an increase in the fresh meat grade, including the quality and safety, since beef is a perishable commodity [2]. The decrease in quality is due to the high fat and water content, making it susceptible to microbial contamination and lipid oxidation.

Proper packaging plays a critical role in preserving the quality and safety of meat. It is critical to select materials that will preserve the packaged product’s qualities and assure its safety. The use of active packaging in conjunction with cold storage is one of the efforts that can be made to prolong the shelf life and enhance the quality.

Active packaging applications can be used to inhibit microbial growth in meat [3,4,5,6,7,8]. These applications incorporate specific active substances into packaging methods to preserve food products by inhibiting bacteria contaminations that contribute to spoilage during storage [9]. The spoilage inhibition mechanism can be conducted in two ways, namely direct and indirect contact. In the direct contact, the packaging materials will preserve food through direct contact, while in the indirect contact, the active packaging releases volatile anti-microbial agents to the packaging system where the food product is placed [10,11]. Camo et al. and Nerin et al. employed an active packaging technique incorporating rosemary and oregano directly touching the meat [12,13], and Campos-Requena et al. placed active packaging containing essential oil to strawberries that was not in direct contact with them [14], and both resulted in a synergistic antibacterial activity.

Volatile anti-microbial compounds are essential oils derived from herbs and spices, such as oregano, thymol, carvacrol, cinnamon, rosemary, ginger, lemongrass, and garlic [15]. Garlic (*allium sativum*) contains the antibacterial component allicin, which is active against both Gram-positive and Gram-negative bacteria [16,17]. The anti-microbial activities of different species of *Allium* have been investigated [18,19,20,21]. Several studies have been conducted on the administration of garlic in active packaging, including the following: Zheng Dong et al. developed an active packaging material based on a PP/LDPE composite film containing *Allium sativum* essential oil. The addition of essential oils into the packaging extends the shelf life of large yellow croaker (*Pseudosciaena crocea*) fillets for 5 days stored at 4 ± 1 °C [22]. Seydim and Sarikus also reported that the anti-microbial properties of whey protein isolate containing essential garlic oil with a ratio of 3.0–4.0% (wt/vol) effectively inhibited the growth of *Salmonella enteritidis*, *S. aureus*, *E. coli* O157:H7, *L. monocytogenes,* and *Lactobacillus plantarum* [23]. The use of garlic extract in active packaging can inhibit microbial growth due to its volatile allicin constituent [24]. Allicin chemicals suppress microbial development by increasing their permeability in penetrating bacterial cell walls to decrease protease enzyme production and disrupt protein and nucleic acid metabolism [19].

The application of active packaging on fresh meat can be accompanied by the addition of intelligent packaging in the form of indicators that monitor changes in quality. This is achieved through chemical reactions between indicators and the results of microbial metabolism or changes in the meat’s chemical composition. Intelligent packaging incorporates indicators that convey information about the product’s quality without causing damage to the packaging and not also in food products. This reduces the risk of loss due to product damage and provides a more accurate condition estimate than expired labels [25,26]. Furthermore, intelligent packaging also assists distributors in adjusting the prices of the products sold [27]. For example, lowering prices when quality has decreased to avoid losing food products. Previous studies applied this method to avocados [28], tuna fillets [29], shrimp [30], cherry tomatoes [31], and *arummanis* mango [32]. Monitoring of color changes can be done due to differences in pH of fresh product and damaged product. According to Shukla et al., color-based pH indicators can be used as intelligent packaging to monitor the presence of volatile amines from microbial metabolites in meat spoilage [33]. The increase in pH during decay causes a significant change in the indicator’s color to be visually observed. Meanwhile, Romero et al. evaluated the quality of packaged cow’s milk using two intelligent packaging prototypes based on the bromothymol blue pH indicator [34]. Julyaningsih et al. and Yolanda et al. combined active and intelligent packaging based on pH indicators methyl red and bromothymol blue to monitor the tuna fillet’s quality [35,36]. Meanwhile, Yong and Liu combined anthocyanin-rich extracts into biopolymer-based films to extend shelf life and monitor the food product’s quality [37].

The spoilage and contamination of food supplies jeopardize global food security. Therefore, the innovative application of active and intelligent packaging needs to be developed. As a result of these considerations, the purpose of this research is to evaluate the efficacy of active paper with garlic extract addition when applied to fresh beef during storage at low temperatures (4 ± 1 °C). Furthermore, it investigates the relationship (correlation) between color analysis of intelligent packaging indicators on various parameters of the beef spoilage test.

## 2. Materials and Methods

### 2.1. Materials

The beef tenderloin was purchased fresh at the slaughterhouse, Tamangapa Raya Antang. Meanwhile, garlic, as an anti-microbial compound in the activated paper, was purchased from supermarkets. Bromothymol blue (Merck, Darmstadt, Germany, CAS No: 76-59-5) and phenol red (Merck, Darmstadt, Germany, CAS No. 34487-61-1) color indicators in intelligent packaging were purchased from Sigma-Aldrich. Mueller Hinton Agar (Oxoid, CM0405) and Nutrient Agar (Merck, Darmstadt, Germany). This research also uses *Escherichia coli* (ATCC 25922), *Staphylococcus aureus* (ATCC 29213), *Pseudomonas aeruginosa* (ATCC 27853), chitosan food grade (YXchuang, Cina), Tween^®^ 80 (Merck, Darmstadt, Germany), corn starch, 96% glacial acetic acid (Brenntag Inc., Essen, Germany), potassium carbonate (K_2_CO_3_) (Merck, Darmstadt, Germany), 7% trichloroacetic acid (TCA) (Merck, Darmstadt, Germany), hydrochloric acid (HCl) (Merck, Darmstadt, Germany), and 0.85% sodium chloride (NaCl) (Merck KGaA, Darmstadt, Germany).

### 2.2. Garlic Extract Preparation

Fifty grams of garlic was blended until smooth and put into a container containing 250 mL of 96% alcohol for a 72 h immersion. During the immersion time, the solution was gently stirred 14 times or every 5 h. Subsequently, the garlic dregs and the filtrate were separated by filtering. At 45 °C, the filtrate was evaporated with an industrial rotary evaporator (TMAX-2L-1) until it became thick reddish-yellow garlic [38].

### 2.3. Active Paper Preparation

Active paper preparation was carried out by cutting Whatman paper no. 1 into small pieces, then 15 g of the small pieces paper was soaked in 250 mL of distilled water for 24 h. The soaked paper was added with 250 mL of distilled water and crushed using a blender for 20 min until a pulp was formed. The paper pulp obtained was squeezed to remove the water, meanwhile 30% (*w*/*w*) tapioca starch was suspended in 50 mL of distilled water, then the pulp and tapioca suspension were homogenized. Furthermore, 100 mL of acetic acid (1%) containing 0.45% chitosan was added to the tapioca and pulp mixture and homogenized with a blender for 5 min. Garlic extract with a concentration according to the treatment (0%, 15% and 20%) (*w*/*w*) was added to the mixture; subsequently, 50 mL of distilled water was also added to the mixture. Furthermore, 0.205 g of Tween^®^ 80 was added and mixed at room temperature using a hotplate magnetic stirrer to generate an emulsion. The paper dough was poured over the surface of the printer container following the styrofoam size used as a beef storage container and flattened to make a wet paper sheet. After that, the wet paper sheet was pressed between the glass surfaces with a load of 2 kg. It was then dried at 40 °C for 48 h, and during the drying process the paper was turned over after 24 h of drying (Modification of Wiastuti et al.) [39].

### 2.4. Indicator Solution Preparation

BTB and PR (1:1) indicator solutions were made by dissolving 1% (*w*/*v*) of the solution in 95% ethanol. Furthermore, the indicator was adjusted to pH 5 using a solution of glacial acetic acid [40].

### 2.5. Intelligent Indicator Label Preparation

Whatman paper no. 1 was cut with a size of 2 × 4 cm and immersed in 20 mL of indicator solution for 12 h at room temperature (28 ± 2 °C). The indicator labels were then dried using an electric dryer (Philips PH 8102) and in a closed container with a drying distance of 30 cm.

### 2.6. Application of Active and Intelligent Packaging on Fresh Beef

**Figure 1:**
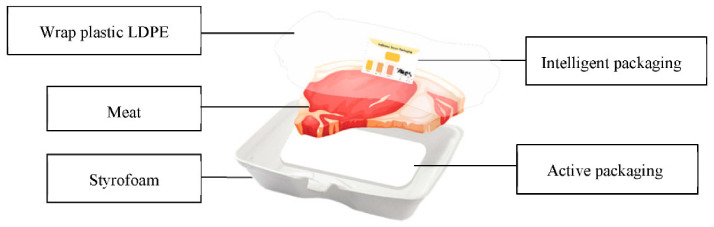
Design of the active packaging and intelligent system.

Fresh beef (tenderloin) with normal pH (5.6–5.7) was obtained from the Tamangapa Antang Makassar abattoir, which was taken at a relative postmortem time of about 3 h. The meat was packaged plastic (PP) containers and put in a cooler box, then immediately transported to the laboratory and prepared sterilely into 180-g pieces/packages. Activated paper with different concentrations was inserted into the styrofoam base. However, since the application of plastic is not sustainable, it is suggested to use more sustainable materials in future. The pieces of meat were packed in styrofoam (1.05 g/cm^3^) and covered with active paper according to the treatment (0%, 15% and 20% of garlic extract) that filled the entire base. The indicator label was placed inside the package by sticking to the LDPE film surface (0.9 g/cm^3^) used as a cover. Observation of beef (tenderloin) packaged with active and intelligent packaging was conducted at a cold temperature (chiller) of 4 ± 1 °C every 3 × 24 h for 21 days of storage. The design for implementing active and intelligent packaging is presented in Figure 1.

### 2.7. Antibacterial Activity Testing of Agar Diffusion Method on Activated Paper

The antibacterial activity of active paper was tested to determine the inhibition toward the growth of Gram-positive bacteria *Staphylococcus aureus*, and Gram-negative bacteria *Escherichia coli* and *Pseudomonas aeruginosa*. Sheets of active paper with a diameter of 5.5 mm were placed on Mueller Hinton Agar (MHA) media, which spread 0.1 mL of the microorganism culture on its surface. Furthermore, the Petri dishes were incubated at 37 °C for 24 h, and after incubation, a clear zone (inhibition zone) was formed around the active paper. The resistance diameter was measured in millimeters (mm) using a caliper [41], and each test was conducted in 3 replications to obtain the average result.

### 2.8. Parameters of Beef Observation Packaged with a Combination of Active and Intelligent Packaging

#### 2.8.1. pH Measurement

The initial and final pH of fresh beef during storage were measured using Horiba Laquatwin Compact pH Meter P-33 with an accuracy of 0.01%. A total of 1 g of the sample was mashed and dissolved in 10 mL of demineralized water. Furthermore, it was inserted on the surface of the pH meter sensor until the value was displayed on the pH meter screen.

#### 2.8.2. TVBN Value Analysis

The meat sample was weighed at 1 g ± 0.1 g, pulverized in a mortar with 3 mL of 7% TCA solution, and filtered to obtain the filtrate. The boric acid solution of 1 mL was put into the “inner chamber” of the conway cup, and the lid was placed in a position to cover the cup. Furthermore, the filtrate was fed into the outer chamber on the left. Then 1 mL of saturated K_2_CO_3_ solution was added to the outer chamber on the right, ensuring that the filtrate and K_2_CO_3_ do not mix. The cup was instantly closed and rotated to mix the two liquids in the outer chamber. The blank solution was prepared the same way as the standard solution, except that the filtrate was replaced with a 7% TCA solution. Subsequently, it was stored at 28 ± 2 °C for 24 h, and the boric acid solution in the inner chamber containing the blank was added with two drops of color indicator solution, then titrated with 0.02 N HCl until it turned pink. The conway cup containing the filtrate was added with two drops of color indicator solution and titrated with the same solution until it turned pink (SNI-2354.8:2009; AOAC, 1995). Equation (1) for determining TVBN:

(1) $$\mathrm{TVBN}\;\mathrm{level}\;\left(\mathrm{mg}/100\;g\right)=\frac{\left(V_{c}-\mathrm{Vb}\right)\times \;N\;\times 14.007\times 100}{w}$$

Description:

Vc = volume of HCl solution in sample titration

Vb = volume of HCl solution in blank titration

N = normality of HCl solution

W = sample weight (g)

14.007 = atomic weight of nitrogen

#### 2.8.3. Calculation of Total Bacterial Count

Total bacteria were calculated based on total plate count method (SNI 2897:2008). The sample solution was made from 1 g of meat homogenized with 9 mL of sterile physiological solution (0.85% NaCl), referred to as the 10^−1^ dilution. Then, the serial dilution was made up to a concentration of 10^−6^. Microbial cultivation using the pour technique in which 1 mL dilutions of 10^−4^, 10^−5^, and 10^−6^ were put into separate sterile petri dishes in duplicate. Subsequently, ±15 mL of sterile NA media was added into the petri dishes, then it was homogenized and incubated at 30 °C for 48 h. Equation (2) for calculation of the total plate count:

(2) $$N=\frac{\Sigma C}{\left[\left(1\times n_{1}\right)+\left(0.1\times n_{2}\right)+\ldots \right]\times \left(D\right)}$$

Description:

N = number of colonies per mL/per gram of product

ΣC = total number of colonies counted

n_1_ = number of cups in the first dilution

n_2_ = number of cups in the second dilution

D = first dilution calculated

### 2.9. Intelligent Indicator Color Measurement Quantification

The color measurement of the intelligent indicator label was analyzed using a digital color meter (T-135). This quantification is conducted by attaching the colorimeter sensor to the intelligent indicator label. The tool will then show the values of L (lightness), a (redness), and b (yellowness) on display. These three values are international standards of color measurement published by the Hunterlab Association Laboratory (2008). Furthermore, the intelligent packaging indicator color is determined by calculating the ^o^Hue value using the Formula (3), and the difference of total color (ΔE) was measured with this Formula (4) [42,43]:

(3) $${}^{\circ}\mathrm{Hue}=\tan^{-1}\frac{b}{a}$$

Description:

°Hue = parameters for color range

a = is a red-green mixed color

b = is a red-green mixed color yellow-blue

(4) $${\Delta E}^{*}=\sqrt{{\left({\Delta L}^{*}\right)}^{2}+{\left({\Delta a}^{*}\right)}^{2}+{\left({\Delta b}^{*}\right)}^{2}}$$

### 2.10. Level of Relationship (Correlation) between Test Parameters

The intelligent packaging indicator quantification data was compared with the data for each beef spoilage parameter presented in a graph using the Sigma Plot software version 14.0.

### 2.11. Statistical Analysis

All data from the test parameters were analyzed using analysis of variance (ANOVA) with Duncan’s multiple range test using SPSS software version 22.0 (IBM Corp., Armonk, NY, USA), and the calculated values differed significantly when *p* < 0.05.

## 3. Results and Discussion

### 3.1. Meat pH

The degree of acidity (pH) in meat is one measure of its freshness, therefore, the analysis of the meat pH value was performed to determine the effect of adding various concentrations of garlic extract during cold storage. Table 1 shows the pH changes that occur in beef during storage.

Table 1 shows that the garlic extract addition with different concentrations affects the increase in pH value of meat during storage. The statistical tests showed that the extract addition on active paper significantly affected the pH value (*p* < 0.05). Further test results showed that the treatment without the garlic extract (0%) was significantly different from the other treatments on average. However, the addition of 15% garlic extract was not significantly different from 20% garlic extract. Ponnampalam et al. stated that the pH of normal meat ranges from 5.5–5.7, and meat with a pH of >6 will be easily damaged by microorganisms, experiencing some deterioration such as color and texture aroma [44]. In Table 1, it can be seen that the initial pH value of fresh meat before storage was 5.68 and categorized as normal, and after storage it was classified as rotten on the 9th days of storage for treatment without garlic extract and on the 15th day for treatment with 15% and 20% garlic extracts.

### 3.2. Total Volatile Bases Nitrogen (TVBN)

The TVBN value is one of the factors used to measure the level of freshness in meat. Table 2 shows the changes in TVBN values in beef during cold storage.

Based on statistical tests, the addition of garlic extract on active paper significantly affected the TVBN value (*p* < 0.05). Further test results showed that the treatment without the extract (0%) addition was significantly different from the other treatments. However, 15% garlic extract was not significantly different from the 20% extract. TVBN criteria for identifying rotten beef products are >20 mg/100 g according to the Ministry of Agriculture and Forestry of Korea, 2016 [45]. The TVBN value of meat on the first day of storage was 4.48 mg N/100, indicating a fresh category (Table 2). On the 9th day of storage, meat treated without the extract had a TVBN value of 27.27 mg N/100 g, categorized as rotten. Meanwhile, the addition of 15% and 20% garlic extract with TVBN values of 22.32 mg N/100 g and 21.66 mg N/100 g had crossed the threshold on day 12. Based on the results obtained, the garlic extract affects the meat TVBN value during storage.

The increase in TVBN value is closely related to the decrease in beef quality. The treatment without garlic extract reached the threshold value earlier due to the higher microbial activity in the sample. The high number of microbes in the sample breaks down more meat protein to produce volatile ammonia compounds. This is consistent with Holman et al., where increased microbial value degrades protein rapidly during storage. Furthermore, it increases the amount of ammonia and other volatile compounds, as well as increases the TVBN value, where these compounds indicate the decay in meat [46]. The increase in the TVBN value of beef with treatment without and with the garlic extract was also different due to the allicin constituent and anti-microbial role. The inhibition of microbial growth minimizes the decomposition process of nutritional components in meat to reduce the TVBN value.

### 3.3. Total Bacteria of Total Plate Count (TPC) Method

The analysis determines the effect of using active paper on the total value of bacteria in meat during cold storage, as shown in Table 3.

The garlic extract added to the active paper had a significant effect on the TPC value of meat (*p* < 0.05). Meanwhile, further test results showed that the treatment without garlic extract (0%) was significantly different from other treatments, but the addition of 15% was not significantly different from the 20% extract. The initial population of TPC meat was 3.52 log CFU/mL, which is categorized as fresh concerning microbiological quality (Table 3). However, this value increased to 6.49 log CFU/mL on the 6th day of storage in the control sample; this value is above the recommended maximum bacteriological limit, which was 6 log CFU/mL (SNI 3932:2008). The meat added with 15% and 20% garlic extract exceeded the maximum bacteriological limit on the 12th day of storage with TPC values of 6.35 log CFU/mL and 6.09 logs CFU/mL, respectively. Therefore, the addition of 15% and 20% extract as active packaging inhibits microbial growth and extends the shelf life of meat up to 6 days longer than the samples without the garlic extract in the active packaging. This is because garlic contains an anti-microbial compound in the form of allicin.

Garlic extract applied to the activated paper diffuses to the entire surface of the beef to inhibit the growth of bacteria through total and partial inhibition of RNA, DNA, and protein synthesis. Furthermore, allicin blocks bacterial enzymes with thiol groups to ultimately inhibit bacterial growth [47]. Mardiya also confirmed that the effectiveness of garlic extract with concentrations of 25%, 50%, and 100% was very effective in inhibiting *Staphylococcus aureus* bacteria and bactericidal [48]. Meanwhile, the extract with a concentration of 12.5% was declared as an inhibitor of bacterial growth (bacteriostatic), but less effective at inhibiting bacteria (bacteriocidal) [48].

The storage temperature of beef can also affect the meat TPC value. Meat cells in the postmortem phase experience metabolic reactions, which are highly dependent on storage temperature. According to Comi, the storage temperature and the metabolic reactions in meat are inversely proportional [49]. This is supported by Kuswandi and Nurfawaidi, where beef stored at room temperature is categorized as unfit for consumption at 10 h with a TPC value of 6.711 CFU/mL. Meanwhile, at cold temperature, it is categorized as unfit for consumption on day 7 with a TPC value of 6.961 CFU/mL [40].

### 3.4. The Antibacterial Analysis Results of Active Paper

**Figure 2:**
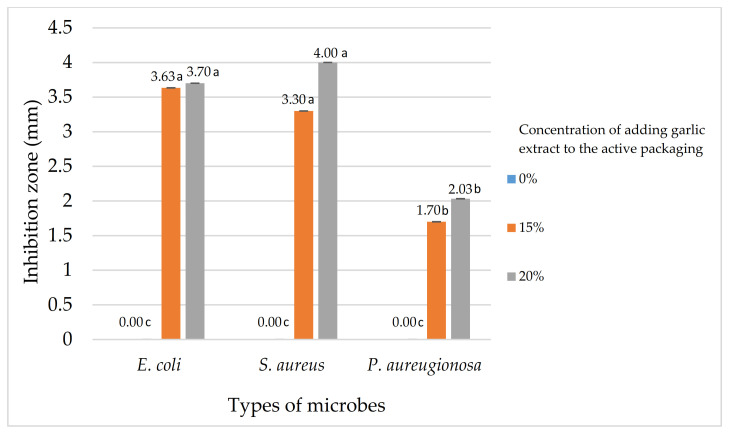
Diameter of bacterial growth barriers on activated paper. The mean value followed by different letters showed a significant difference based on the Tuckey’s test at the 5% level (*p*-value < 0.05).

Antibacterial activity on active paper was tested against several bacteria that generally cause damage to beef, namely *Escherichia coli*, *Staphylococcus aureus*, and *Pseudomonas aeruginosa*. Figure 2 illustrates the active paper’s analytical findings for the bacterial inhibition zone region.

It can be seen from Figure 2 that with no addition of garlic extract to active paper (0%), the paper shows no inhibition zones to all three strains of bacteria tested. In comparison to active paper with the addition of both 15% and 20% garlic extract, it displayed bacterial inhibitory activity, implying that garlic extract possessed antibacterial activity.

According to Pan et al., the response to bacterial growth inhibition was divided into three categories: antibacterial activity with zones of inhibition greater than 6.00 mm (strong), 3–5 mm (moderate), and less than 3 mm (weak) [50]. Figure 2 provides information that shows active packaging with no addition of garlic extract had no inhibitory activity to all bacteria tested. While the addition of 15% and 20% of garlic extract to the active packaging showed a moderate response against *E. coli* (3.6 mm and 3.3 mm, respectively) and also moderate against *S. aureus* (3.7 mm and 4 mm, respectively). While for *P. aeruginosa* the active packaging categorized has a weak inhibitory response both in the administration of 15% and 20% garlic extract with 1.7 mm and 2 mm, respectively.

The diameter of the inhibitory zone created against *Escherichia coli*, *Stapylococcus aureus*, and *Pseudomonas aeruginosa* bacteria varies and could be due to the differences in bacterial structure that affect the penetration of allicin compounds through bacterial cell walls. The inhibitory zone formed by active packaging with the addition of both 15% and 20% garlic extract was classified as weak against *P. aeruginosa* due to the fact that gram-negative bacteria such as *P. aeruginosa* have complex cell walls, which prevent antibacterial compounds from penetrating the bacteria cell sufficiently to inhibit bacterial growth [51]. However, the findings of this study indicated that the application of garlic extract to the active package had the same inhibitory zone category against *E.coli* and *S.aureus*, even though *E. coli* also includes Gram-negative bacteria. Even though the previous explanation for *P. aeruginosa* does not support the result obtained in this research for *E. coli*, these findings are consistent with the findings of Garba et al. and Safithri et al., who discovered that the inhibitory action to *E.coli* bacteria was typically more or equal to that of *S. aureus* [52,53].

### 3.5. Color Change Response on Intelligent Indicator Labels for Beef Packaged in Active Packaging at Cold Temperature Storage

**Figure 3:**
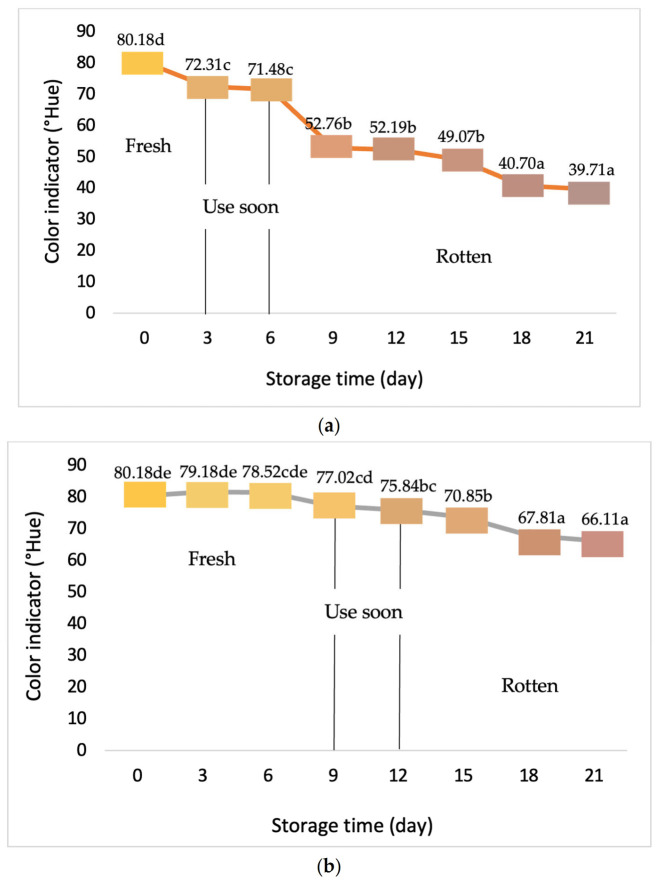

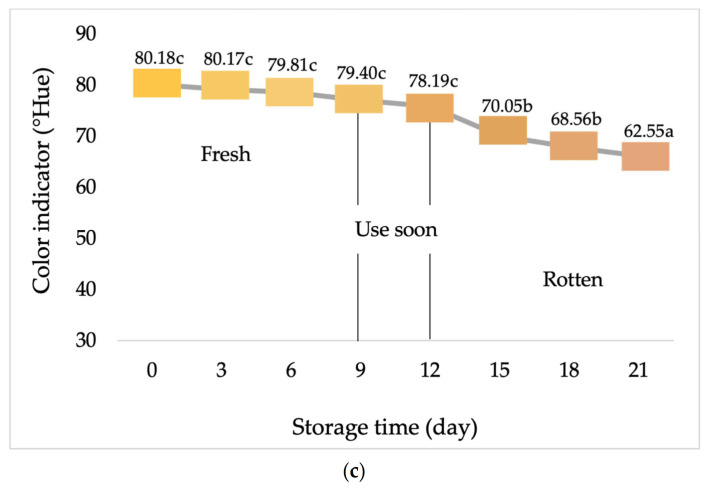
Color change profile of intelligent indicator labels with garlic extract addition on the concentrations at 0% (**a**), 15% (**b**), and 20% (**c**). The mean value followed by different letters showed a significant difference based on the Duncan’s test at the 5% level (*p*-value < 0.05). Phase I with dark yellow indicating fresh, phase II with yellow and red gradations indicating have to be consumed soon, and phase III with faded red indicating rotten meat.

The color change analysis of the intelligent indicator determines the effect of adding garlic extract to active packaging with different concentrations on beef during storage at cold temperatures (chiller). The color change of the resulting indicator label is information of the freshness condition of the packaged beef. Significant color changes on indicators ease consumers to measure the freshness level based on visual appearance, as seen in Figure 3.

Figure 3 showed that the ^o^Hue value on the intelligent indicator label decreased during storage, from dark yellow to faded red. The color change is a response to the quality condition. There are three phases of change on intelligent packaging labels in the description. 

The color change on the intelligent indicator label is closely related to the increased number of microbes and enzymes in meat during storage. These changes are caused by the decomposition of nutritional components of meat by enzymes and microbes, producing volatile base compounds used as an early sign of damage in packaged beef. This is consistent with Sani et al. Who showed that volatile ammonia compounds in meat can increase the pH [54]. The increase in the pH of meat is caused by ammonia reacting with H+ ions to produce H ions. The OH- ions in the packaging are directly proportional to the pH value, affecting the change in intelligent indicator labels.

**Figure 4:**
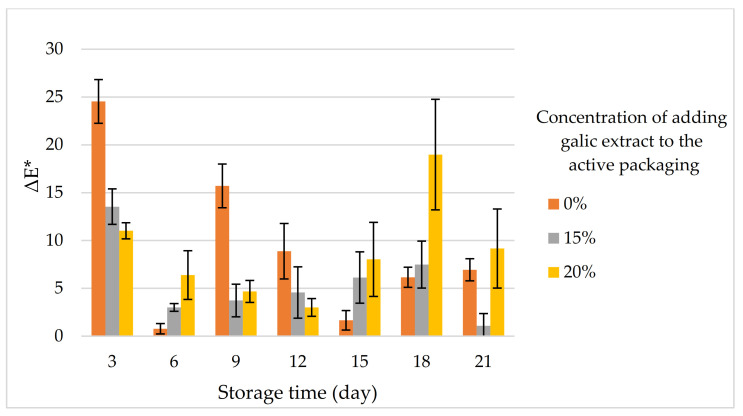
Total color difference (ΔE^*^).

Delta E is a standard measurement for quantifying the difference between two colors. The standard delta E perception consists of five scales: 1.0 is invisible to the human eye; 1–2 visible through observation; 2–10 at a glance; 11–49 colors are more similar than opposite; and 100 colors are opposite [55]. Based on Figure 4, the smart indicator shows the value of delta E with a range of 1–18 for each treatment, except on storage days 3–6, the value of delta E is 0.77. This shows that smart indicators are generally easy to visualize based on color changes in smart packaging systems. Based on this, consumers will easily make visual assessments and also monitor the freshness of the meat through intelligent packaging sensors.

### 3.6. Correlation of Color Value Changes in Intelligent Packaging Indicators BTB: PR (1:1) with the Effect of Active Packaging on All Meat Spoilage Parameters

The relationship between the color change response of intelligent indicator labels with all test parameters on meat, such as TVBN, TPC, and pH, determined the sensitivity of intelligent indicator labels in detecting quality deterioration. This obtains synchronization between changes in the color of the intelligent indicator label to the deterioration of meat quality.

**Figure 5:**
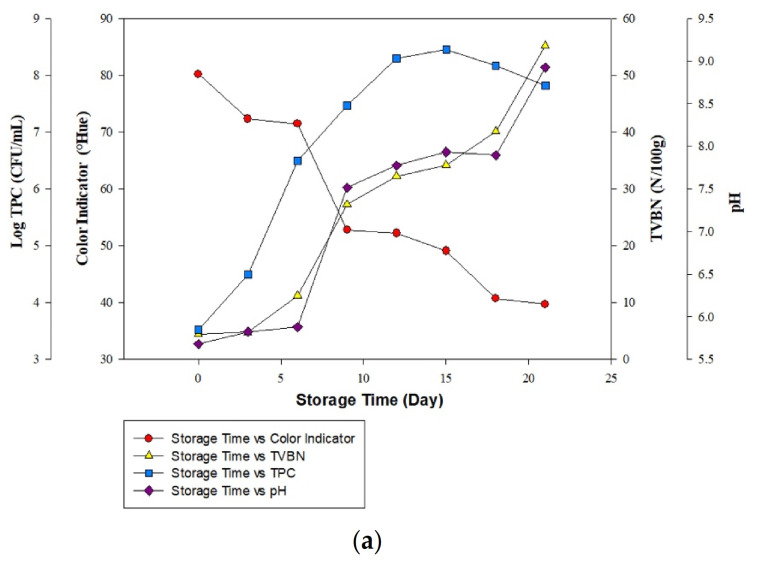

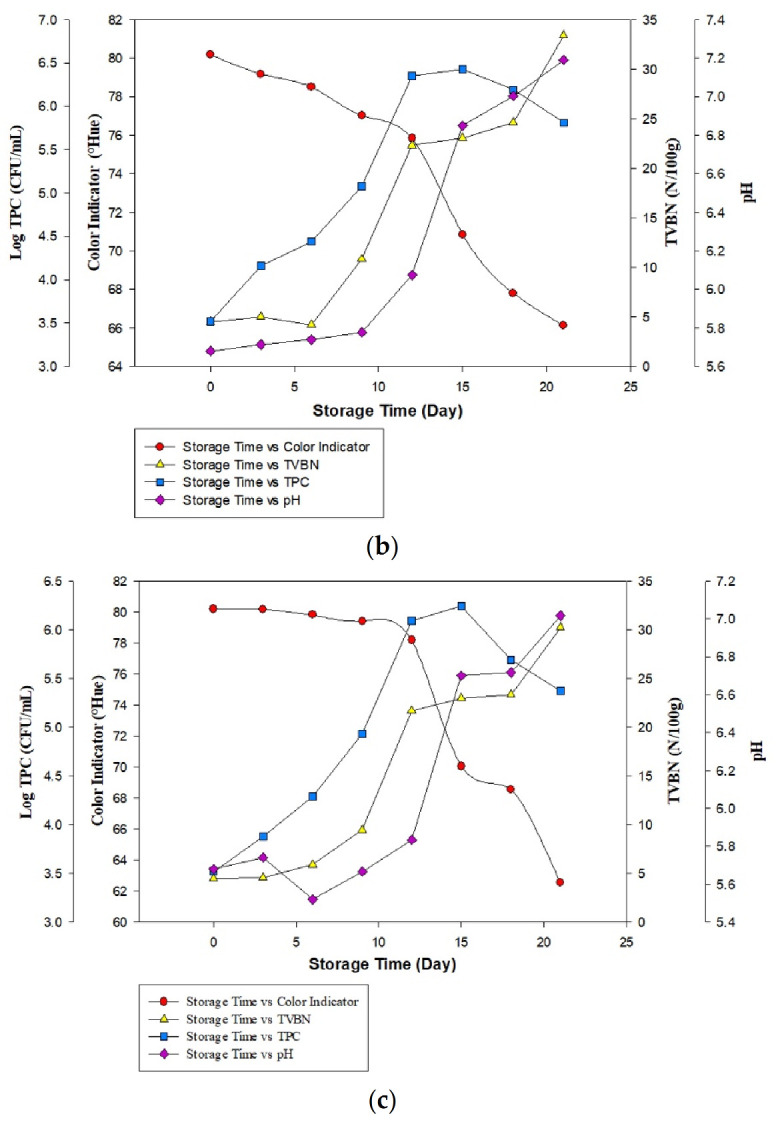
Correlation of color change on intelligent indicator label with all beef spoilage parameters with the garlic extract addition: (**a**) control, (**b**) 15%, and (**c**) 20%.

Based on Figure 5, beef stored without the garlic extract (0%) showed that the increase in the TVBN value and the pH of the meat was in line with the decrease in the color value of the intelligent packaging indicator. However, it is not consistent with the results of the TPC value since the beef TPC value crossed the threshold on the 6th day of storage, which is 6.4 CFU/mL. Meanwhile, the TVBN value is suitable for consumption on the 6th day. Mansur et al. also reported that the TVB-N values were inconsistent with the viable microbial count (TVC) [56]. Based on TVB-N, the samples were classified as damaged after 7 and 11 days of storage [56]. This is because the microbial calculation method is not selective against spoilage bacteria in meat but counts all the pathogenic microbes. Holman et al. also stated that the TVB-N value was more precise and accurate in classifying beef in fresh or rotten conditions than the total microbial value (TVC) [46].

Based on Figure 5b,c, beef stored with 15% and 20% garlic extract treatment showed that the increase in the parameters of the meat quality deterioration, including TPC, TVBN, and pH, was consistent with the decrease in the color of the intelligent indicator label. The intelligent indicator changes color from yellow (fresh) to faint red (rotten) during storage. The increase in TVBN, TPC, and pH values is influenced by the high nutritional content, specifically protein broken down into polypeptides and other amino acids through the deamination process to form ammonia. With the increase in microbes, the nutritional components decomposed by microbes are also greater and produce basic volatile compounds, increasing the pH value of meat during storage. The resulting volatile compounds accumulate in the packaging and are directly detected by the intelligent indicator label. The reduction in the color value of the intelligent indicator label shows the presence of many alkaline volatile chemicals in the package and the rottenness and unfitness for the ingestion of the meat. Furthermore, when viewed for the visual appearance on the 6th and 12th day of storage without (0%) and with (15% and 20%) garlic extract, the beef was damaged and unfit for consumption, which is characterized by the formation of mucus, emitting a sour/rotten smell, and the texture beginning to be sticky to the touch.

The increase in the beef quality deterioration parameter value for each treatment was different due to the addition of garlic extract as active packaging. The extract has a bioactive compound in allicin to inhibit microbial growth. This compound in the package diffuses in the meat surface to suppress the microbe growth. According to Deresse, it works by completely inhibiting bacterial RNA synthesis and inhibiting DNA and protein synthesis [47]. The pattern of relationship between the parameters of the decline in beef quality and the color analysis value of indicator labels is also consistent with Kuswandi and Nurfawaidi, where a linear relationship pattern to the parameters of the beef freshness level with changes in the color intensity of the bromocresol purple and methyl red intelligent labels was observed [40]. In this study, beef treated without the garlic extract experienced a decrease in quality and was not suitable for consumption on the 6th day. Meanwhile, with 15% and 20% garlic extract, the meat was damaged and unfit for consumption on the 12th day of storage. Previous research applying garlic extract as active packaging in meat was performed and resulted as the 15% garlic extract enhancing the shelf life of meat to 16 h from 12 h without garlic extract at room temperature [57]. This indicates that garlic extract storing at cold temperatures can extend the shelf life of packaged meat. Visually, the intelligent indicator label used Whatman paper no. 1 as the base material, and the BTB + PR (pH 5.00) used in intelligent packaging for beef freshness, with a color change from yellow (beginning) to faint red (late). Therefore, it assists customers in determining freshness without touching the texture or opening the packaging.

## 4. Conclusions

The garlic extract in active packaging, supported by the microbiological evaluation results of TVBN and pH, extended shelf life during storage. In addition, the results showed that the active paper with 15% and 20% garlic extract extended the shelf life to 12 days of cold storage. The color change profile of the intelligent indicator label combination of BTB + PR pH 5.00 was easily observed from dark (fresh) to reddish yellow (to be consumed immediately). The red color fades when the beef is rotten and unfit for consumption. Therefore, the combination of active and intelligent packaging is suggested as a potential packaging application in fresh meat packaging.

## Figures and Tables

**Table 1:** The pH value of packaged meat samples stored in cold storage for 21 days.

Storage Time (Day)	Addition of Garlic Extract on Active Paper		
	0%	15%	20%
0	5.68 ± 0.06 ^g^	5.68 ± 0.06 ^g^	5.68 ± 0.06 ^g^
3	5.82 ± 0.16 ^g^	5.71 ± 0.01 ^g^	5.74 ± 0.16 ^g^
6	5.88 ± 0.0 ^g^	5.74 ± 0.01 ^g^	5.52 ± 0.16 ^g^
9	7.52 ± 0.72 ^b,c,d^	5.78 ± 0.07 ^g^	5.67 ± 0.16 ^g^
12	7.77 ± 0.52 ^b,c^	6.08 ± 0.01 ^f,g^	5.83 ± 0.13 ^g^
15	7.93 ± 0.20 ^b^	6.85 ± 1.27 ^d,e,f^	6.70 ± 0.62 ^e,f^
18	7.90 ± 0.13 ^b^	7.00 ± 0.66 ^b,c,d,e^	6.72 ± 0.03 ^e,f^
21	8.93 ± 0.14 ^a^	7.19 ± 0.58 ^b,c,d,e^	7.02 ± 0.60 ^c,d,e^
Average	7.18 ± 1.22 ^A^	6.25 ± 0.65 ^B^	6.11 ± 1.35 ^B^

The mean value followed by different letters showed a significant difference based on the Duncan’s test at the 5% level (*p*-value < 0.05).

**Table 2:** The TVBN value of packaged meat samples stored in cold storage for 21 days.

Storage Time (Day)	Addition of Garlic Extract on Active Paper		
	0%	15%	20%
0	4.48 ± 0.00 ^i^	4.48 ± 0.00 ^i^	4.48 ± 0.00 ^i^
3	4.76 ± 0.00 ^i^	5.04 ± 1.40 ^i^	4.58 ± 1.38 ^i^
6	11.21 ± 1.46 ^g^	4.20 ± 0.00 ^i^	5.88 ± 0.56 ^h,i^
9	27.27 ± 2.72 ^d,e^	10.83 ± 0.65 ^g^	9.43 ± 0.32 ^g,h^
12	32.22 ± 9.54 ^c^	22.32 ± 1.38 ^f^	21.66 ± 2.83 ^f^
15	34.18 ± 1.40 ^c^	23.06 ± 0.71 ^f^	22.97 ± 1.22 ^f^
18	40.81 ± 1.17 ^b^	24.65 ± 1.84 ^e,f^	23.35 ± 1.71 ^e,f^
21	55.28 ± 4.66 ^a^	33.43 ± 1.13 ^c^	30.26 ± 1.28 ^c,d^
Average	26.27 ± 18.19 ^A^	16.00 ± 11.26 ^B^	15.33 ± 10.30 ^B^

The mean value followed by different letters showed a significant difference based on the Duncan’s test at the 5% level (*p*-value < 0.05).

**Table 3:** The TPC value of packaged meat samples stored in cold storage for 21 days.

Storage Time (Day)	Addition of Garlic Extract on Active Paper		
	0%	15%	20%
0	3.52 ± 0.05 ^i^	3.52 ± 0.05 ^i^	3.52 ± 0.05 ^i^
3	4.49 ± 0.04 ^f,g,h^	4.16 ± 0.08 ^g,h,i^	3.88 ± 0.06 ^h,i^
6	6.49 ± 0.06 ^c^	4.44 ± 0.03 ^f,g,h^	4.29 ± 0.18 ^g,h,i^
9	7.47 ± 0.02 ^b^	5.08 ± 0.07 ^e,f^	4.93 ± 0.48 ^f,g^
12	8.30 ± 0.04 ^a^	6.35 ± 0.71 ^c,d^	6.09 ± 0.20 ^c,d^
15	8.45 ± 1.22 ^a,b^	6.43 ± 1.27 ^c,d,e^	6.24 ± 0.69 ^c,d^
18	8.17 ± 0.61 ^a,b^	6.19 ± 0.15 ^c,d^	5.69 ± 0.41 ^d,e^
21	7.82 ± 0.16 ^a,b^	5.81 ± 0.24 ^c,d,e^	5.37 ± 0.54 ^e,f^
Average	6.84 ± 1.87 ^A^	5.25 ± 1.11 ^B^	5.00 ± 1.02 ^B^

The mean value followed by different letters showed a significant difference based on the Duncan’s test at the 5% level (*p*-value < 0.05).

## Data Availability

Available data are presented in the manuscript.

## References

[1] Dave D., Ghaly A.E. (2011). Meat spoilage mechanisms and preservation techniques: A critical review. Am. J. Agric. Biol. Sci..

[2] Yu H.H., Chin Y.-W., Paik H.-D. (2021). Application of Natural Preservatives for Meat and Meat Products against Food-Borne Pathogens and Spoilage Bacteria: A Review. Foods.

[3] Djenane D., Gómez D., Yangüela J., Roncalés P., Ariño A. (2019). Olive Leaves Extract from Algerian Oleaster (*Olea europaea* var. sylvestris) on Microbiological Safety and Shelf-life Stability of Raw Halal Minced Beef during Display. Foods.

[4] Bahmid N.A., Dekker M., Fogliano V., Heising J. (2021). Development of a moisture-activated antimicrobial film containing ground mustard seeds and its application on meat in active packaging system. Food Packag. Shelf Life.

[5] Umaraw P., Munekata P.E.S., Verma A.K., Barba F.J., Singh V.P., Kumar P., Lorenzo J.M. (2020). Edible films/coating with tailored properties for active packaging of meat, fish and derived products. Trends Food Sci. Technol..

[6] Alizadeh-Sani M., Mohammadian E., McClements D.J. (2020). Eco-friendly active packaging consisting of nanostructured biopolymer matrix reinforced with TiO_2_ and essential oil: Application for preservation of refrigerated meat. Food Chem..

[7] Lin L., Luo C., Li C., Chen X., Cui H. (2022). Application in Beef Preservation. Foods.

[8] Kapetanakou A.E., Pateraki G.-L., Skandamis P.N. (2020). Developing a Commercial Antimicrobial Active Packaging System of Ground Beef Based on “Tsipouro” Alcoholic Distillate. Foods.

[9] Arkoun M., Daigle F., Holley R.A., Heuzey M.C., Ajji A. (2018). Chitosan-based nanofibers as bioactive meat packaging materials. Packag. Technol. Sci..

[10] Quintavalla S., Vicini L. (2002). Antimicrobial food packaging in meat industry. Meat Sci..

[11] Yildirim S., Röcker B., Pettersen M.K., Nilsen-Nygaard J., Ayhan Z., Rutkaite R., Radusin T., Suminska P., Marcos B., Coma V. (2018). Active Packaging Applications for Food. Compr. Rev. Food Sci. Food Saf..

[12] Camo J., Beltrán J.A., Roncalés P. (2008). Extension of the display life of lamb with an antioxidant active packaging. Meat Sci..

[13] Nerín C., Tovar L., Djenane D., Camo J., Salafranca J., Beltrán J.A., Roncalés P. (2006). Stabilization of beef meat by a new active packaging containing natural antioxidants. J. Agric. Food Chem..

[14] Campos-Requena V.H., Rivas B.L., Pérez M.A., Figueroa C.R., Sanfuentes E.A. (2015). The synergistic antimicrobial effect of carvacrol and thymol in clay/polymer nanocomposite films over strawberry gray mold. LWT-Food Sci. Technol..

[15] Drago E., Campardelli R., Pettinato M., Perego P. (2020). Innovations in Smart Packaging Concepts for Food: An Extensive Review. Foods.

[16] Putnik P., Gabrić D., Roohinejad S., Barba F.J., Granato D., Mallikarjunan K., Lorenzo J.M., Bursać Kovačević D. (2019). An overview of organosulfur compounds from *Allium* spp.: From processing and preservation to evaluation of their bioavailability, antimicrobial, and anti-inflammatory properties. Food Chem..

[17] Shang A., Cao S.-Y., Xu X.-Y., Gan R.-Y., Tang G.-Y., Corke H., Mavumengwana V., Li H.-B. (2019). Bioactive Compounds and Biological Functions of Garlic (*Allium sativum* L.). Foods.

[18] Santas J., Almajano M.P., Carbó R. (2010). Antimicrobial and antioxidant activity of crude onion (*Allium cepa*, L.) extracts. Int. J. Food Sci. Technol..

[19] Kyung K.H. (2012). Antimicrobial properties of allium species. Curr. Opin. Biotechnol..

[20] Radusin T., Torres-Giner S., Stupar A., Ristic I., Miletic A., Novakovic A., Lagaron J.M. (2019). Preparation, characterization and antimicrobial properties of electrospun polylactide films containing *Allium ursinum* L. extract. Food Packag. Shelf Life.

[21] Angane M., Swift S., Huang K., Butts C.A. (2022). Essential Oils and Their Major Components: An Updated Review on Antimicrobial Activities, Mechanism of Action and Their Potential Application in the Food Industry. Foods.

[22] Dong Z., Luo C., Guo Y., Ahmed I., Pavase T.R., Lv L., Li Z., Lin H. (2019). Characterization of new active packaging based on PP/LDPE composite films containing attapulgite loaded with *Allium sativum* essence oil and its application for large yellow croaker (*Pseudosciaena crocea*) fillets. Food Packag. Shelf Life.

[23] Seydim A.C., Sarikus G. (2006). Antimicrobial activity of whey protein based edible films incorporated with oregano, rosemary and garlic essential oils. Food Res. Int..

[24] Yolanda D.S., Dirpan A., Rahman A.N.F., Kamaruddin I., Ainani A.F. (2020). Determination the best concentration of antimicrobial ingredients with a mixture of paper to create active paper packaging. IOP Conf. Ser. Earth Environ. Sci..

[25] Lee S.J., Rahman A.T.M.M. (2013). Intelligent Packaging for Food Products.

[26] Pacquit A., Crowley K., Diamond D., Kerry J. (2008). Smart Packaging Technologies for Fish and Seafood Products. Smart Packaging Technologies for Fast Moving Consumer Goods.

[27] Vilas C., Mauricio-Iglesias M., García M.R. (2020). Model-based design of smart active packaging systems with antimicrobial activity. Food Packag. Shelf Life.

[28] Dirpan A., Djalal M., Rahman R., Genisa J. (2021). The utilization of red cabbage extract (*Brassica oleracea*) in the production of avocado (*Persea americana Mill*) freshness indicator as smart packaging element. Online J. Biol. Sci..

[29] Agustianti D., Dirpan A., Syarifuddin A. (2021). The potential application of red cabbage indicator film as smart packaging on tuna fillet. IOP Conf. Ser. Earth Environ. Sci..

[30] Wu Y., Tang P., Quan S., Zhang H., Wang K., Liu J. (2021). Preparation, characterization and application of smart packaging films based on locust bean gum/polyvinyl alcohol blend and betacyanins from cockscomb (*Celosia cristata* L.) flower. Int. J. Biol. Macromol..

[31] Kim D., Thanakkasaranee S., Lee K., Sadeghi K., Seo J. (2021). Smart packaging with temperature-dependent gas permeability maintains the quality of cherry tomatoes. Food Biosci..

[32] Dirpan A., Latief R., Syarifuddin A., Rahman A.N.F., Putra R.P., Hidayat S.H. (2018). The use of colour indicator as a smart packaging system for evaluating mangoes Arummanis (*Mangifera indica* L. var. Arummanisa) freshness. IOP Conf. Ser. Earth Environ. Sci..

[33] Shukla V., Kandeepan G., Vishnuraj M.R. (2015). Development of On-Package Indicator Sensor for Real-Time Monitoring of Buffalo Meat Quality During Refrigeration Storage. Food Anal. Methods.

[34] Romero A., Sharp J.L., Dawson P.L., Darby D., Cooksey K. (2021). Evaluation of two intelligent packaging prototypes with a pH indicator to determine spoilage of cow milk. Food Packag. Shelf Life.

[35] Julyaningsih A.H., Latief R., Dirpan A. (2020). The making of smart and active packaging on tuna fillet. IOP Conf. Ser. Earth Environ. Sci..

[36] Sisilia Yolanda D., Dirpan A., Nur Faidah Rahman A., Djalal M., Hatul Hidayat S. (2020). The potential combination of smart and active packaging in one packaging system in improving and maintaining the quality of fish. Canrea J. Food Technol. Nutr. Culin. J..

[37] Yong H., Liu J. (2020). Recent advances in the preparation, physical and functional properties, and applications of anthocyanins-based active and intelligent packaging films. Food Packag. Shelf Life.

[38] Gosal L., Hutomo S., Sooai C.M. (2021). Garlic (*Allium sativum* L.) Ethanolic Extract Capability to Inhibit Pseudomonas aeruginosa Biofilm Formation. J. Med. Health.

[39] Wiastuti T., Khasanah L.U., Kawiji W.A., Manuhara G.J., Utami R. (2016). Characterization of active paper packaging incorporated with ginger pulp oleoresin. IOP Conf. Ser. Mater. Sci. Eng..

[40] Kuswandi B., Nurfawaidi A. (2017). On-package dual sensors label based on pH indicators for real-time monitoring of beef freshness. Food Control.

[41] Mohammed M.F., Raman N., Alhoot M.A., Alwan M.R. (2020). Antibacterial activities of allium sativum (Garlic) extracts against staphylococcus aureus and *Escherichia coli*. Eur. J. Mol. Clin. Med..

[42] Hunterlab (2008). Colorimeters Versus Spectrophotometers.

[43] Hernández-García E., Vargas M., Torres-Giner S. (2022). Quality and shelf-life stability of pork meat fillets packaged in multilayer polylactide films. Foods.

[44] Ponnampalam E.N., Hopkins D.L., Bruce H., Li D., Baldi G., El-din Bekhit A. (2017). Causes and Contributing Factors to “Dark Cutting” Meat: Current Trends and Future Directions: A Review. Compr. Rev. Food Sci. Food Saf..

[45] Korean Ministry of Agriculture and Forestry. https://www.mfds.go.kr/eng/brd/m_15/view.do?seq=70016.

[46] Holman B.W.B., Bekhit A.E.D.A., Waller M., Bailes K.L., Kerr M.J., Hopkins D.L. (2021). The association between total volatile basic nitrogen (TVB-N) concentration and other biomarkers of quality and spoilage for vacuum packaged beef. Meat Sci..

[47] Deresse D. (2010). Antibacterial effect of garlic (*Allium sativum*) on Staphylococcu aureus: An in vitro study. Asian J. Med. Sci..

[48] Mardiyah S. (2018). Efektivitas Anti Bakteri Perasan Bawang Putih (*Allium sativum* L.) terhadap Pertumbuhan Staphylococcus aureus. Med. (J. Med. Lab. Sci.).

[49] Comi G. (2017). Spoilage of Meat and Fish.

[50] Pan X., Chen F., Wu T., Tang H., Zhao Z. (2009). The acid, bile tolerance and antimicrobial property of Lactobacillus acidophilus NIT. Food Control.

[51] Costa A.L.R., de Oliveira A.C.S., Azevedo V.M., Medeiros E.A.A., Soares N.d.F.F., Borges S.V. (2020). Essential oils of garlic and oregano incorporated in cellulose acetate films: Antimicrobial activity and physical properties. Res. Soc. Dev..

[52] Garba I., Umar A., Abdulrahman A., Tijjani M., Aliyu M., Zango U., Muhammad A. (2014). Phytochemical and antibacterial properties of garlic extracts. Bayero J. Pure Appl. Sci..

[53] Safithri M., Bintang M., Poeloengan M. (2011). Antibacterial activity of garlic extract against some pathogenic animal bacteria. Media Peternak..

[54] Alizadeh-Sani M., Tavassoli M., Mohammadian E., Ehsani A., Khaniki G.J., Priyadarshi R., Rhim J.W. (2021). PH-responsive color indicator films based on methylcellulose/chitosan nanofiber and barberry anthocyanins for real-time monitoring of meat freshness. Int. J. Biol. Macromol..

[55] Schuessler Z. What Is Delta E? and Why Is It Important for Color Accuracy?. http://zschuessler.github.io/DeltaE/learn/.

[56] Mansur A.R., Song E.J., Cho Y.S., Nam Y.D., Choi Y.S., Kim D.O., Seo D.H., Nam T.G. (2019). Comparative evaluation of spoilage-related bacterial diversity and metabolite profiles in chilled beef stored under air and vacuum packaging. Food Microbiol..

[57] Dirpan A., Djalal M., Kamaruddin I. (2022). Application of an intelligent sensor and active packaging system based on the bacterial cellulose of Acetobacter xylinum to meat products. Sensors.

